# Characterization of the Shells in Layer-By-Layer Nanofunctionalized Particles: A Computational Study

**DOI:** 10.3389/fbioe.2022.888944

**Published:** 2022-06-30

**Authors:** E. Barchiesi, T. Wareing, L. Desmond, A. N. Phan, P. Gentile, G. Pontrelli

**Affiliations:** ^1^ Instituto de Investigación Cientifica, Universidad de Lima, Lima, Peru; ^2^ École Nationale d’Ingénieurs de Brest, Brest, France; ^3^ School of Engineering, Newcastle University, Newcastle Upon Tyne, United Kingdom; ^4^ Istituto per le Applicazioni del Calcolo-CNR, Rome, Italy

**Keywords:** drug release, layer-by-layer, mathematical modeling, drug diffusion, drug dissolution

## Abstract

Drug delivery carriers are considered an encouraging approach for the localized treatment of disease with minimum effect on the surrounding tissue. Particularly, layer-by-layer releasing particles have gained increasing interest for their ability to develop multifunctional systems able to control the release of one or more therapeutical drugs and biomolecules. Although experimental methods can offer the opportunity to establish cause and effect relationships, the data collection can be excessively expensive or/and time-consuming. For a better understanding of the impact of different design conditions on the drug-kinetics and release profile, properly designed mathematical models can be greatly beneficial. In this work, we develop a continuum-scale mathematical model to evaluate the transport and release of a drug from a microparticle based on an inner core covered by a polymeric shell. The present mathematical model includes the dissolution and diffusion of the drug and accounts for a mechanism that takes into consideration the drug biomolecules entrapped into the polymeric shell. We test a sensitivity analysis to evaluate the influence of changing the model conditions on the total system behavior. To prove the effectiveness of this proposed model, we consider the specific application of antibacterial treatment and calibrate the model against the data of the release profile for an antibiotic drug, metronidazole. The results of the numerical simulation show that ∼85% of the drug is released in 230 h, and its release is characterized by two regimes where the drug dissolves, diffuses, and travels the external shell layer at a shorter time, while the drug is released from the shell to the surrounding medium at a longer time. Within the sensitivity analysis, the outer layer diffusivity is more significant than the value of diffusivity in the core, and the increase of the dissolution parameters causes an initial burst release of the drug. Finally, changing the shape of the particle to an ellipse produces an increased percentage of drugs released with an unchanged release time.

## 1 Introduction

Polymeric microparticles (MPs) are receiving increasing interest as drug delivery systems (Karp et al., 2019) and in tissue engineering and biosensing (Qodratnama et al., 2015; Martins et al., 2018) due to their advantages such as the ease of manufacturing and potential industrial scale up (Chung et al., 2019). Furthermore, if applied as drug delivery systems, MPs presents interesting features such as the opportunity to use different administration routes or encapsulate various bioactive molecules, including nucleic acid and proteins (McKiernan et al., 2018; Ospina-Villa et al., 2019).

The release of the drug payload can be easily tuned by selecting the polymer and its chemical characteristics, such as monomer composition, molecular weight, glass transition temperature, and crystallinity (Takeuchi et al., 2018; Lagreca et al., 2020). Another important aspect is the selection of the manufacturing method that can provide the MPs with specific morphological properties, size, and polydispersity index. These characteristics are fundamental to guarantee stability, encapsulation, and suitable drug release (Busatto et al., 2018). Thus, manufacturing techniques need to be selected according to the drug payload and specific MP applications (Wang et al., 2017). However, MPs have some limitations including the presence of organic solvent residues, tendency to form aggregates, high burst release, and instability of encapsulated bioactive molecules (Lagreca et al., 2020). Therefore, there is an urgent need for advanced technologies to obtain coated MPs that enable addressing these challenges, allowing localized and controlled release of the active therapeutics (Lavan et al., 2003; Park et al., 2018). Strategies for manufacturing of thin and homogenous films account for the Langmuir–Blodgett technique (Park et al., 2011), self-assembled monolayer methods (Mani et al., 2008), and layer-by-layer (LbL) assembly (Gentile et al., 2015; Gentile et al., 2017). The latter technique is most suited for fabrication of films that can be applied as drug delivery systems due to their versatility with no limitations on the shape and size of the substrate and requires mild processing conditions, without using high pressure and temperature.

Furthermore, LbL coatings are deposited onto the substrate surface by alternating the adsorption of the charged materials, such as polyelectrolytes (PEs), proteins, graphene oxide, and micelles (Park et al., 2018). The interaction of these charged materials involves driving forces such as electrostatic potential, covalent or hydrogen bonds, and biological interactions. The polymers used for the LbL assembly of MPs mainly are mainly synthetic PEs, such as poly(allylamine hydrochloride) (PAH) (Gentile et al., 2015), polyethyleneimine (PEI) (Li et al., 2011; Ju et al., 2016), poly (styrene sulfonate) (PSS) (Park et al., 2011), and poly(acrylic acid) (PAA) (Xie et al., 2014; Ju et al., 2016). However, the use of natural-based PEs is considered an attractive alternative for biomedical applications due to their biomimetic and improved biological properties (Sood et al., 2021). The selection of the materials can influence the thickness of the multilayer. Particularly, synthetic polymers are considered mainly strong PEs, and they present higher values (∼15–25 nm of thickness for each layer) at the same concentration values as the natural ones (few-15 nm) (Grant et al., 2001). Furthermore, LbL assembly can be useful for high payload and sustained release as shown by Szczęsna et al. (2021), where they manufactured multilayered alginate/chitosan microparticles for encapsulating biomolecules.

With reference to the release aspect, mathematical and computational *(in silico*) modeling offers an alternative tool in drug delivery design and provides more insight on the effects of various geometrical and physical conditions that can be useful to drastically reduce the number of experiments and, also, to predictive screening instrument for the design and manufacturing of drug-loaded systems (Siepmann and Siepmann, 2012). Within this field, the mathematical models can be divided into two large groups, that is, mechanistic or empirical/semi-empirical. The latter consists typically of quite simple equations that can be easily managed by the experimentalists. A large number of these models (Peppas, Weibull, Gompertz, et al.) have been proposed over the years and are based on a simple power–law relationship involving time and drug release, with the exponent that represents the mechanism of release (e.g., diffusion, erosion, swelling or non-Fickian diffusion) (Ritger and Peppas, 1987). The fully mechanistic continuum models, which have been gaining increasing attention, are based on different phenomena *via* more difficult physics-based equations and accounting for parameters having a direct physical and chemical meaning (Pontrelli et al., 2021). However, each strategy is characterized not only by advantages but also by limitations and challenges. In our work, we develop a continuum-scale mathematical model to evaluate the transport and release of a drug from an MP. This model accounts for two different components (core and shell) and considers the dissolution and diffusion of the drug into the core and, also, the diffusion and the retention of the drug into the shell. After the identification of the relevant parameters over a set of *in vitro* experiments, we perform sensitivity analysis to understand the influence of different model conditions on the full system behavior. We show that the model, after assessment through a procedure of optimization, is capable of fitting the observed experimental data and, thus, can be used as a predictive tool.

## 2 Modeling Drug Release From a Multilayer Particle

One of the main advantages of LbL assembly consists in the opportunity to modulate and control the release of the drug by modifying the number of layers, and therefore, the MP final thickness. Particularly, two growth regimes are reported: 1) the linear regime involves a coating growing linearly with each PE deposition step that only appears if the charge of the new PE overcompensates the surface charge of the previous later upon, and 2) the nonlinear regime (i.e., exponential) that depends mainly on the polymeric dynamics of multilayered coating and involves a thickness increasing exponentially with each PE deposition step (Campbell and Vikulina, 2020). Several works in literature have reported that a minimum number of layers (from four nanolayers or two bi-layers) are required in order to successfully change the properties of the substrate surface (Radtchenko et al., 2000; Pontremoli et al., 2021). This involves the formation of precursor layers that enable decreasing possible interference from the substrate to get a better charged uniform surface and, ultimately, a more homogenous and stable multilayer (Peng et al., 2012). Furthermore, the PE charge has also a fundamental role in the formation of a stable multilayer, where two charge compensation types are commonly reported: intrinsic and extrinsic charge compensation. The former accounts for the charge balance between the previous PEs into the multilayer, while the latter occurs when extra PE charge is compensated *via* salt ions present in the solution (i.e., Na^+^ and Cl^−^) (Ghoussoub et al., 2018). However, during the growth of the multilayer, a saturation stage, mainly managed by the electrostatic interaction between the dissolved PEs in the solvent and the progressively neutralized substrate surface, is reached and does not allow any more deposition of layers (Zhang et al., 2018).

**FIGURE 1:**
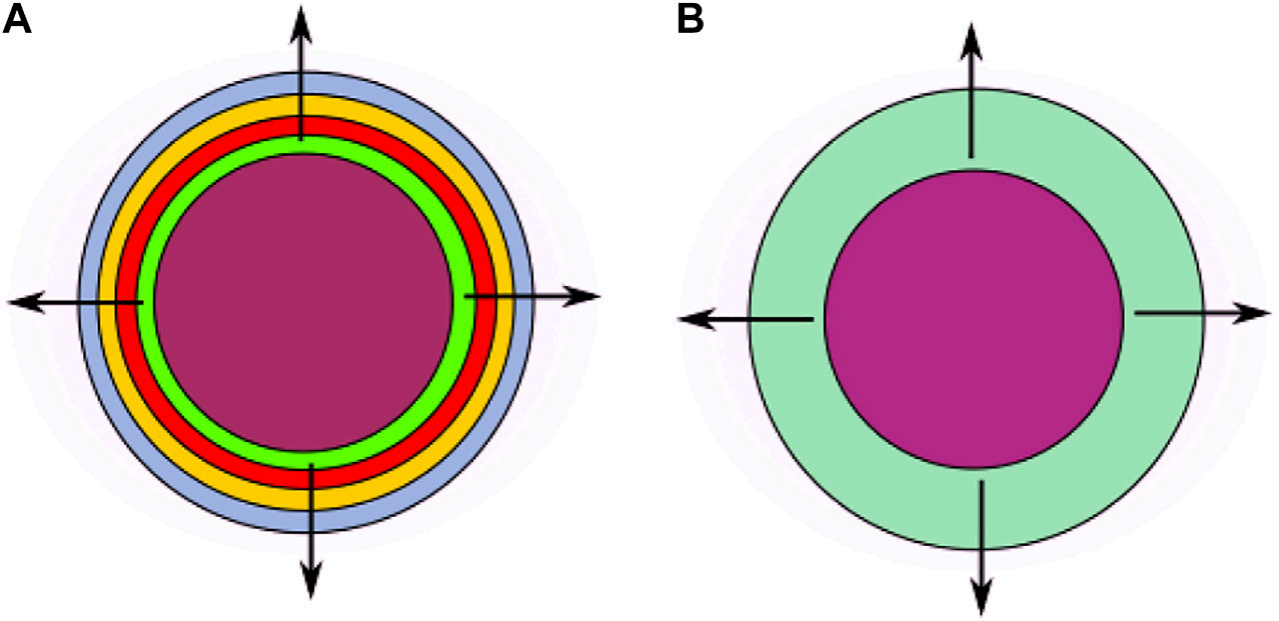
Diagram of the cross section of a spherical two-layer system based on an internal core and concentric LbL coating (left, **(A)**). With reference to their releasing properties, the enveloping layers can be assimilated to a unique equivalent shell having averaged characteristics (right, **(B)**) (figures not to scale).

**FIGURE 2:**
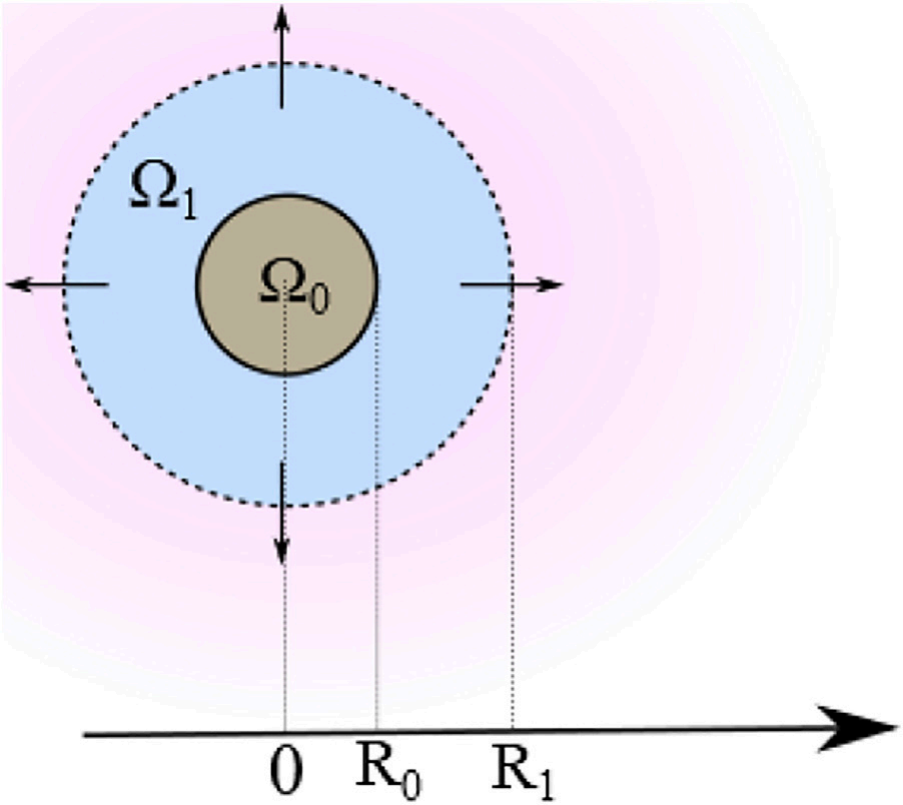
Schematic of the 2D cross-section of a multilayer system comprising an internal core Ω_0_ and LbL-equivalent external shell Ω_1_ (figure not to scale).

To model drug release from MPs, we consider a multilayer spherical system that accounts for an internal core $\Omega_{0}$ and *n* enveloping concentric layers *Ω*_*1*_, *Ω*_*2*_, .. *Ω*_*n*_. These layers have, all together, a thickness much smaller than the radius of the core but offer a significant resistance to the drug flux. Most drugs are entrapped into the internal core, and we consider an initial homogeneous distribution at some concentration *B*_*0*_. However, the MP assembly configuration can determine that a certain amount of drug can initially be entrapped in the other layers. We consider that this drug remains permanently encapsulated and will not be released. For the purposes of sole release, however, we can model the multiple shells with a unique equivalent external layer having averaged characteristics and yielding the same cumulative releasing effect (Figure 1). Thus, the resulting two-layer MP comprises a spherical core *Ω*_*0*_ of radius *R*_*0*_ and an enveloping shell *Ω*_1_ of radius *R*_*1*_ protecting the core and covering the MP surface. Let us consider a coordinate system with the origin located at the center of the sphere and the r-axis oriented with the positive direction pointing outward (Figure 2).

Regarding *in vitro* experiments, when exposed to the release medium, the particle uptakes surrounding liquid (phosphate buffer saline, PBS) and a mechanism of dissolution results in the filled core, transforming the immobile (undissolved) drug with concentration *b*_*0*_(***x**, t*) to dissolved drug with concentration *c*_*0*_(***x***, *t*). As we have demonstrated previously (Pontrelli et al., 2021), we assume model dissolution as a nonlinear process where the drug entrapped into the core can dissolve at a specific rate *β* proportionally to the difference between the concentration of dissolved drug and its solubility *S* in the physiological solution. After dissolution, the drug is capable of diffusing over the core with a diffusion coefficient $D_{0}$ . The drug dissolution and diffusion dynamics in $\Omega_{0}$ are defined by the nonlinear partial differential equations:

$$\frac{\partial b_{0}}{\partial t}=-\beta b_{0}^{\alpha }\left(S-c_{0}\right),$$

$$\frac{\partial c_{0}}{\partial t}=\nabla \cdot \left(D_{0}\nabla c_{0}\right)+\beta b_{0}^{\alpha }\left(S-c_{0}\right),\;\mathrm{in}\;\Omega_{0}$$ (2.1)

where ∇ stands for the gradient operator. The α exponent takes into consideration possible effects on the dissolution rate because the particle surface area varies. Typically, in the case of spherical particles, α=23 (Frenning, 2003).

Furthermore, in view of modeling drug kinetics in the shell, experiments show that due to electrostatic interactions, a low percentage of the initial loaded drug is mainly kept without any release (Toniolo et al., 2018). We model this aspect by using first-order reaction kinetics, where diffusing the drug through the shell can potentially be permanently bound with a rate *k* (Pontrelli et al., 2021). Although other forms of reaction/interaction can be considered, in the current work, we concentrate our investigation on a simple linear reaction model. Representing the bound and unbound phase concentrations by *b*_*1*_ *(* ***x*** *, t)* and *c*_*1*_ *(* ***x*** *, t)* respectively, the dynamics of the drug in $\Omega_{1}$ can be described by the equations (Pontrelli et al., 2021):

$$\frac{\partial c_{1}}{\partial t}=\nabla \cdot \left(D_{1}\nabla c_{1}\right)-kc_{1},$$

$$\frac{\partial b_{1}}{\partial t}=+kc_{1},\;\mathrm{in}\;\Omega_{1}.$$ (2.2)

where $D_{1}$ is the diffusion coefficient in the coating shell.

To close the system Eqs 2.1, 2.2, proper initial and boundary conditions are imposed. At the interfaces between the core and shell, we consider continuity of normal flux and concentration:

$$-D_{0}\nabla c_{0}\cdot n=-D_{1}\nabla c_{1}\cdot n,\;c_{0}=c_{1}\;at\partial \Omega_{0}\cap^{\text{​}}\;\partial \Omega_{1}.$$ (2.3)

At the outer surface, mimicking the *in vitro* experimental conditions where the MP is immersed in a large environment fluid, we impose a perfect sink condition:

$$c_{1}=0\mathrm{at}\;\partial {\text{Ω}}_{1}.$$ (2.4)

At initial time, the concentration of the undissolved loaded drug in the core is *B*_*0*_, and the bound drug concentration in the shell is *B*_*1*_:

$$b_{0}=B_{0},\;c_{0}=0,\;b_{1}=B_{1},\;c_{1}=0.$$ (2.5)

At any time, the drug total mass present in the MP is specified by integrating the concentration of each layer over the corresponding volume (Pontrelli et al., 2021), as

$$M_{tot}\left(t\right)=\int_{{\text{Ω}}_{0}}\left\{b_{0}\left(x,t\right)+c_{0}\left(x,t\right)\right\}dx+\int_{{\text{Ω}}_{1}}\left\{b_{1}\left(x,t\right)+c_{1}\left(x,t\right)\right\}dx.$$ (2.6)

The release profile, $M_{rel}\left(t\right)$ , is determined as the cumulative % of drugs released by time t and can be defined as

$$\%M_{rel}\left(t\right)=\frac{M_{tot}\left(0\right)-M_{tot}\left(t\right)}{M_{tot}\left(0\right)}\times 100,$$ (2.7)

where $M_{tot}\left(0\right)$ represents the total initial drug mass. A relevant performance indicator will be release time determined as the minimum time *t*_*r*_ beyond which $\%M_{rel}\left(t\right)$ has a small variation (<0.1%) (Pontrelli et al., 2020).

## 3 Experimental Set up and Release Considerations

The manufacture of the LbL-functionalized MP is a complex procedure where several factors need to be considered within the experimental setup for efficient incorporation and release of a drug from the multilayer structure. Initially, the possible toxicity of the biomolecules and of the polyelectrolytes needs to be evaluated before the manufacturing of the LbL multilayer coating, incorporating the selected drug (Li et al., 2017). The second aspect is MP stability, where the enveloping layers entrapping drugs need to be physically and chemically stable (Goldberg et al., 2007). To improve it, the number of layers produced, variety of interactions, and crosslinking chemistry need to be carefully considered. Particularly, a high number of layers can increase the stability and the homogeneity of the prepared multilayer (Amancha et al., 2014; Szczęsna et al., 2021). Another important aspect is the drug release rate that should be devised to ensure a sustained rather than burst release, taking into consideration the target site and the dose. This can be accomplished by tuning the rate of film degradation rate that depends on the material, the fabrication method, and the type of drug incorporated. Furthermore, the controllable wall permeability between the core and the shell, which represents a fundamental LbL MP characteristic, makes their use encouraging for drug delivery (Szczęsna et al., 2021). By using this feature, different drugs have been entrapped into the hollow MPs by tuning easily the surface permeability at various salt concentrations or pH (Johnston et al., 2006), though, the application of this procedure is limited to the MP formulations or/and the loaded drugs, and the drug entrapment percentage can be very low as the maximum drug concentration in the MP is frequently restricted to the concentration in the physiological solution (Mao et al., 2005).

**FIGURE 3:**
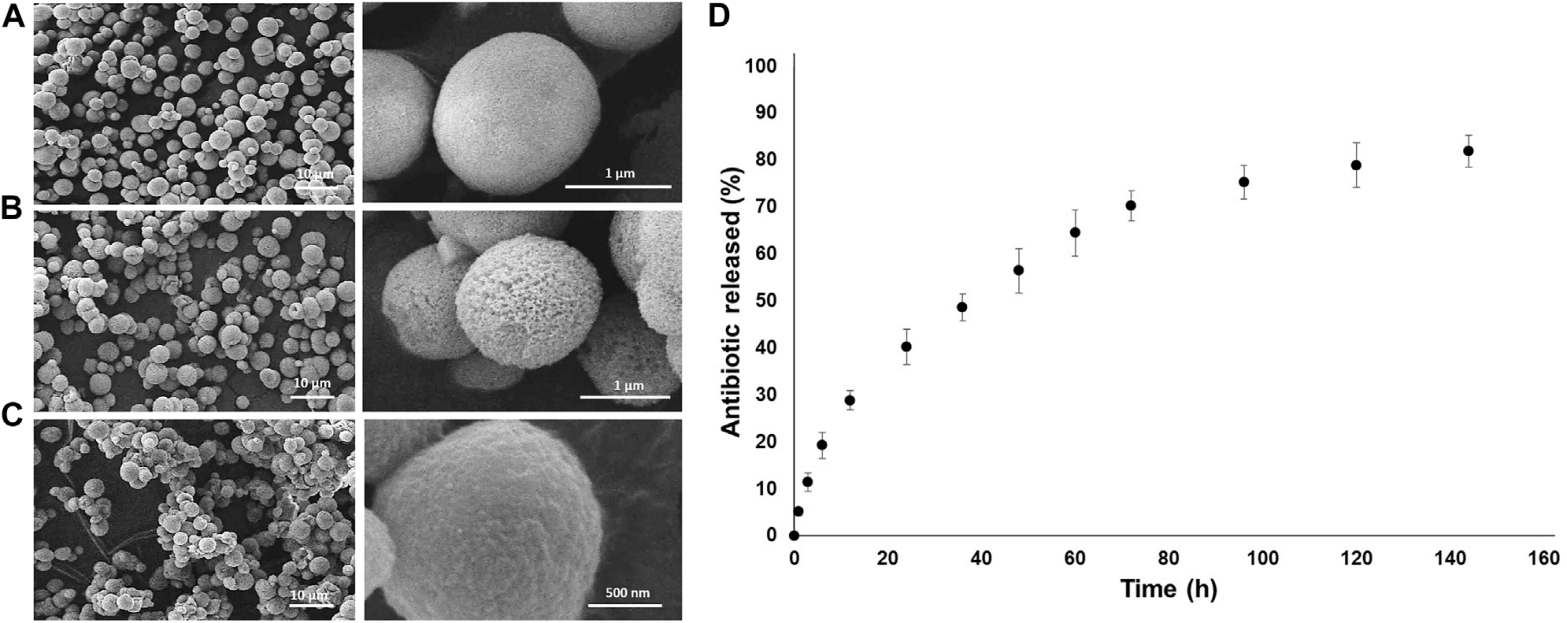
SEM Images of the MP as prepared **(A)**, after eight layers **(B)** and after 14 layers **(C)**. *In vitro* release data from the LbL-functionalized MPs **(D)**. Each data point represents the mean ± standard deviation over a set of six tested samples.

**FIGURE 4:**
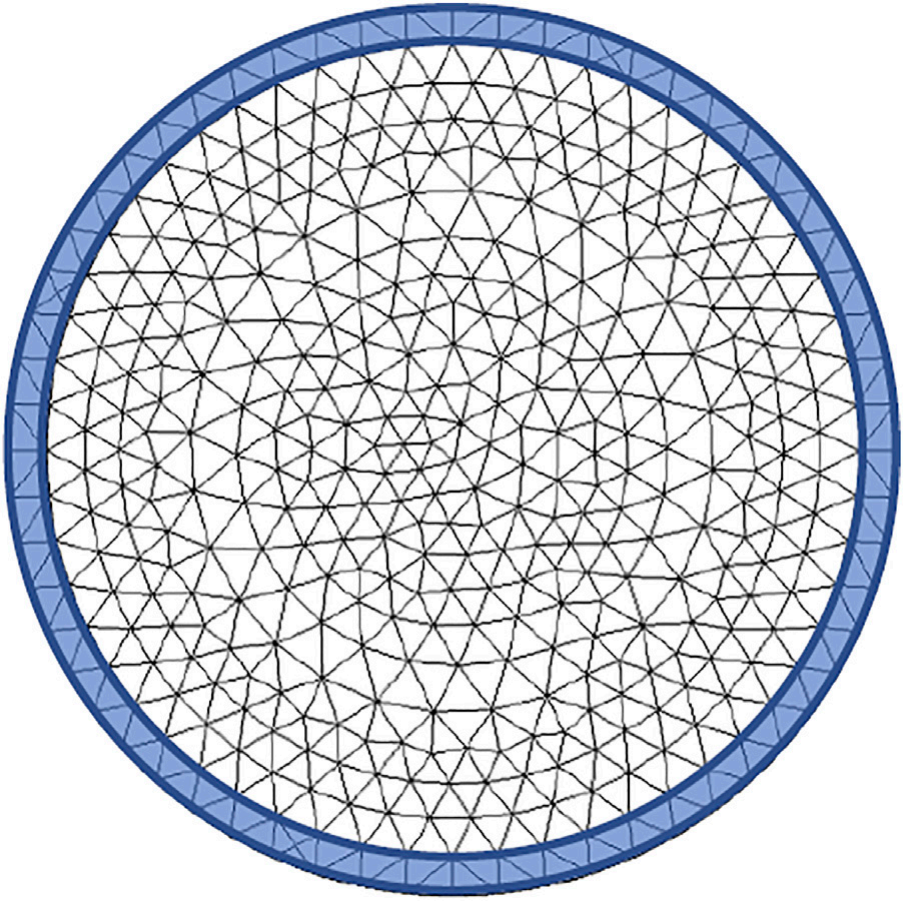
FEM mesh in the two-layer spherical particle.

For the manufacturing of drug-loaded MPs, calcium carbonate (CaCO_3_) cores were synthesized according to the protocol reported by Volodkin et al. (2004) by using CaCl_2_ and Na_2_CO_3_ as precursors at the same molarity, 0.33 M, and with the same volume. PSS (1 mg/ml) was dissolved in Na_2_CO_3_ in order to prevent the cuboidal calcite crystalline phase of CaCO_3_. The reagents were initially solubilized in deionized water (dH_2_O) separately, and the solution containing Na_2_CO_3_ and PSS was rapidly poured into the CaCl_2_ solution under constant and intense mixing, over a magnetic hot plate at room temperature (RT) for 10 min, centrifuged three times in dH_2_O in order to separate the CaCO_3_ from the supernatant and, then, left to dry at RT. For the LbL multilayered formation, aqueous solutions of both PEs (PSS and PAH) were prepared in 0.5 M NaCl at 5 mg/ml of concentration. The washing steps were carried out in 0.05 M NaCl water solution, and the pH of all the solutions was titrated at 6.5. The CaCO_3_ MPs, characterized by a negative ζ-potential (−20.1 ± 0.4 mV), were dissolved in the PE solutions (10 ml per 100 mg of CaCO_3_), starting with the polycation (PAH) for 15 min of immersion; then three washing steps by centrifuging the MPs for 2 min at 1200 rpm were carried out to remove the excess PE. The process was repeated to create 14 layers (seven cycles). The metronidazole (MT), a drug with high antimicrobial activity against anerobic bacteria and protozoa, was loaded into the CaCO_3_ core by prior dissolution in the CaCl_2_ reagent. After the LbL procedure, the obtained functionalized MPs were dried at 37°C in an oven and then stored at 3°C. The growth of the multilayered coating formation was observed by scanning electron microscopy (SEM) analysis (Figure 3). After eight layers, the LbL-MP surface looked very rough and porous (Figure 4B), while, after the deposition of the 14 nanolayers, a more homogeneous and smoother coated surface was observed (Figure 4C). By using ImageJ software, after the LbL functionalization, the diameter of the bare MPs (CaCO_3_ only) increased from an average diameter of 1.39 ± 0.43 µm to 1.52 ± 0.32 μm. Furthermore, the successful multilayer coating manufacturing was confirmed by ζ-potential measurements and infrared spectroscopy analysis using FT IR-ATR (see Supplementary Figures S1, S2). *In vitro* MT release, after incubation of the LbL MPs at 37°C, was calculated by UV–Vis spectrophotometry and shown in Figure 4D. The MT release profile showed triphasic behavior: an early linear trend with approximately 20% of the released drug within the first 12 h and then a controlled and sustained MT release (70–80%), before reaching a plateau after 140 h. These release results are suitable for the application of multilayered loaded MPs that are intended to improve the properties over a 7-day period when bacterial infections occur *in vivo*. Information on the methods used for the MP characterization is reported in the Supplementary Material.

## 4 Numerical Simulation and Results

The drug release mechanism from a multilayer MP modeled by Eqs 2.1, 2.2 depend on a large number of interdependent parameters, whose value cannot often be identified empirically. Furthermore, even when some of these unknown parameters are obtainable from published works, they can be frequently subjected to high uncertainty and inconsistency. Thus, having available reliable parameter estimates represents an important issue in this area.

In this two-dimensional computational study, the geometrical and physical parameters are chosen to simulate the release from a coated core-shell drug-loaded particle and in agreement with typical values and release times from experiments as in the previous section (Figure 3). The size (diameters) of the MP layers has been selected as follows:

$$d_{0}^{*}=1.39\;\mu m\;,\;d_{1}^{*}=1.52\;\mu m.$$ (4.1)

That results in the following volumes (areas in 2D), respectively:

$$V_{0}=1.5175\;\mu m^{2}\;,\;V_{1}=0.2971\;\mu m^{2}.$$ (4.2)

The core has been initially loaded by the encapsulated drug, with the enveloping shell free of the drug, and the respective masses are as follows:

$$M_{0}=2.5\;\cdot 10^{-4}\mu g\;,\;M_{1}=0.$$ (4.3)

To solve the mathematical problem in Eqs 2.1, 2.2, and in view of more complex geometries, we employ the finite element method by using COMSOL Multiphysics software (COMSOL Multiphysics, 2019). The FEM triangular mesh has a maximum size of 0.16 µm which guarantees a convenient resolution. Further refining of the mesh did not alter the solution (grid independence) (Figure 4). The simulation has been run over 400 h, with a discretization time step automatically adapted.

**FIGURE 5:**
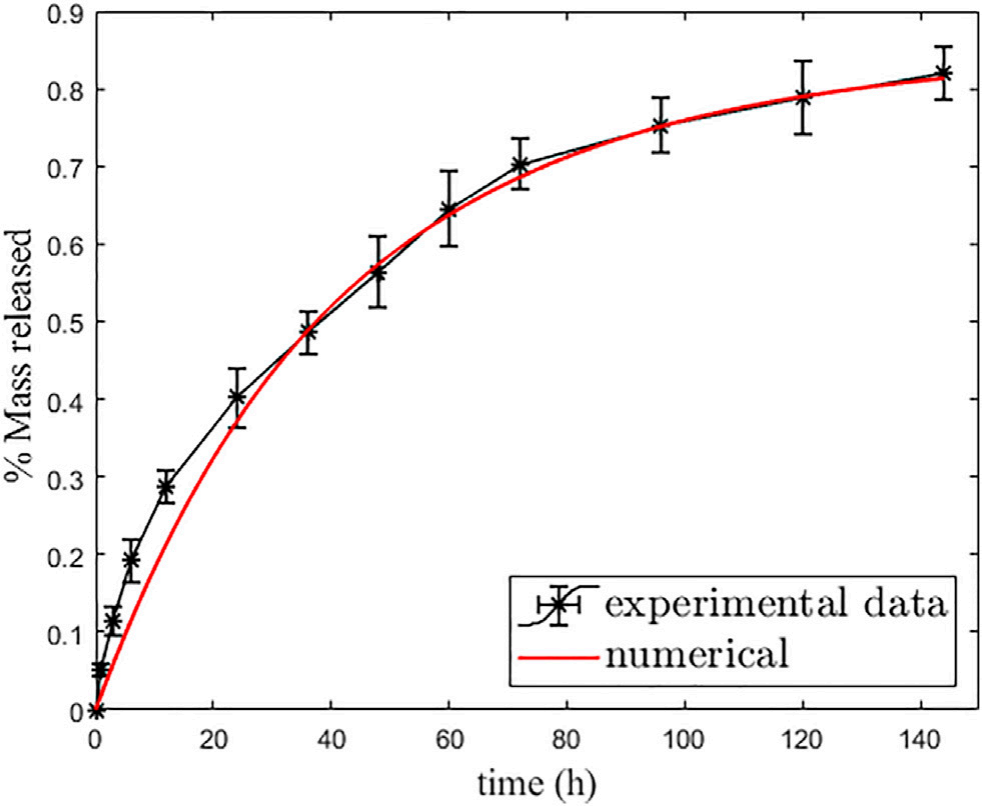
Best fit curve of simulation (red line) over experimental data (black line with error bars).

It is known that the shell acts as a selective barrier for drug release, with a consequent reduction of mobility to prevent fast release. In the external environment, the mass accumulates gradually, influenced by the material characteristics of the two layers. So, because of the absorbing condition (Eq. 2.4), the overall drug mass is transported to the external release medium at an adequately extended time. In Table 1, we report the six model parameters that have been identified by best fit over the experimental data, and Figure 5 shows the correspondent cumulative percentage of mass released (release curve). It appears that about 85% of the drug is released (with a release time of about 233 h), while 15% remains permanently bound in the MP. The drug release appears well-described by the developed two-layer dissolution–diffusion-reaction model.

**TABLE 1:** Reference parameters (denoted by *) as obtained by the best fit in the present case study.

Parameter	Value (units)
$D_{0}^{*}$	$2.5\cdot 10^{-10}cm^{2}s^{-1}$
$D_{1}^{*}$	$2.5\cdot 10^{-11}cm^{2}s^{-1}$
$\alpha^{*}$	1.1
$\beta^{*}$	$2.7\cdot 10^{-6}s^{-1}{\left(g\;cm^{-2}\right)}^{-\alpha }$
$S^{*}$	$0.03\;g\;cm^{-2}$
$k^{*}$	$0.2\;s^{-1}$

**FIGURE 6:**
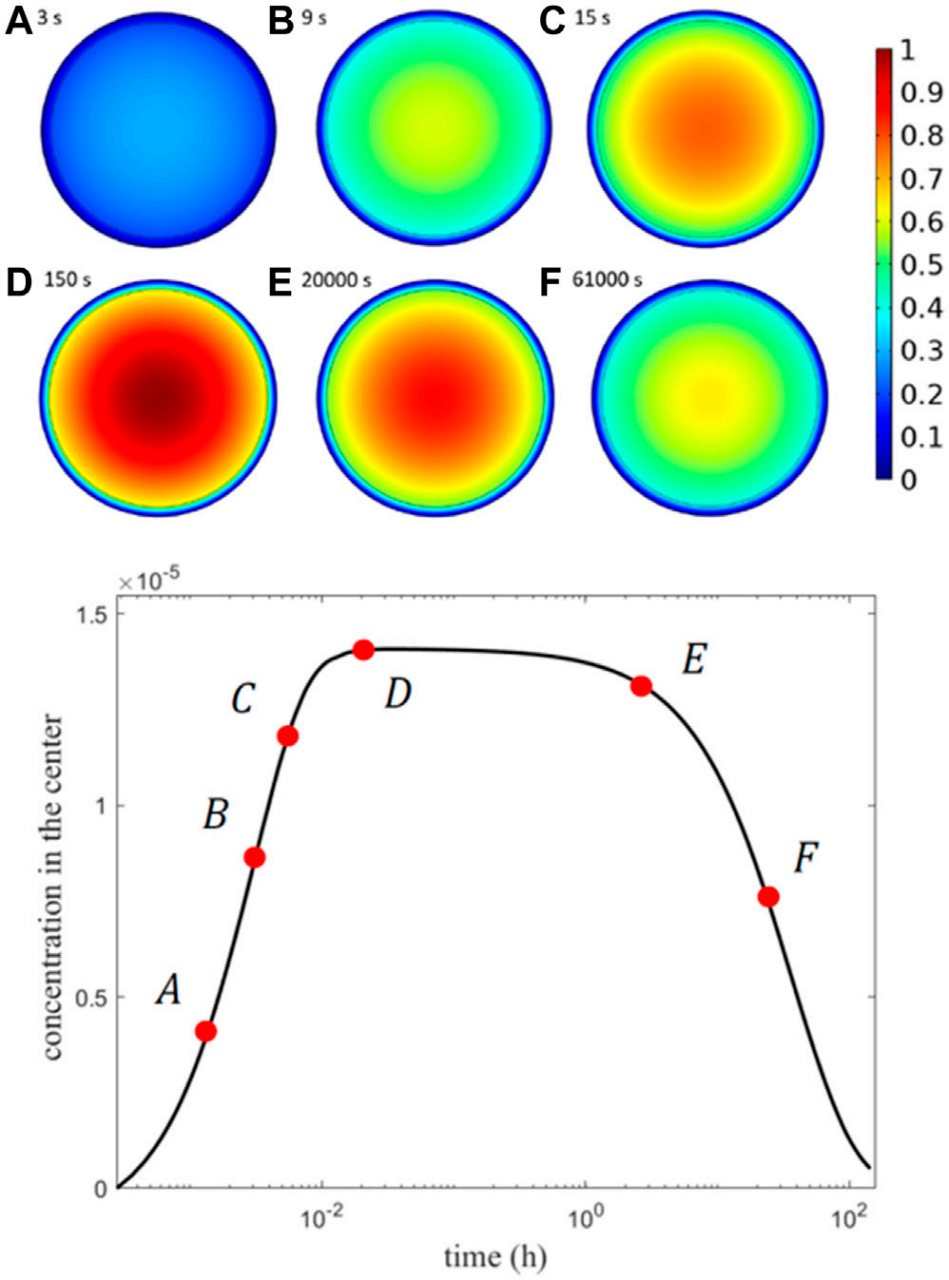
Top: concentration field at six different times labelled from A (after 3 s) to F (after 61000 s). Bottom: plot of the maximum concentration at the MP center vs. time at the 6 selected times.

In Figure 6, the concentration field at six different time points is displayed: two regimes are easily recognizable, in the first, shorter, the drug dissolves, diffuses in the core, and travels to the external shell layer; in the second longer regime, the drug is finally transported from the shell to the release medium at a sufficiently extended time. Due to its high resistance, drug kinetics is much slower in the shell, until it is released externally at a rate that depends on the diffusive properties. The influence of the joint multi-diffusivity is comparable to that of other existing drug release carriers (Kaoui et al., 2018; Pontrelli et al., 2021). The thin outer shell retains a negligible mass because of its thickness, and the core is totally emptied after an incubation time of approximately 16 days, when all the mass is moved to the shell and released to the surrounding environment. In the case of smaller diffusivity coefficients, a much-sustained release takes place.

### 4.1 Sensitivity Analysis

We now investigate the sensitivity of the model to the variation with respect to the case study, when a parameter at a time is varied. In particular, we concentrate our study on the properties of the shell, when its material parameters are changed. Then, we proceed also to study the variation of the solution with respect to the shape, in particular when flattening of the particle occurs, and the sphere becomes an ellipse.

#### 4.1.1 Influence of Diffusivity

We now consider a variation in the properties of the MP material. This has been parameterized first by the different ratios of core diffusivity with respect to the reference case being in the range:

$$\frac{D_{0}}{D_{0}^{*}}=\;10^{-4}-10^{4}.\;$$ (4.4)

**FIGURE 7:**
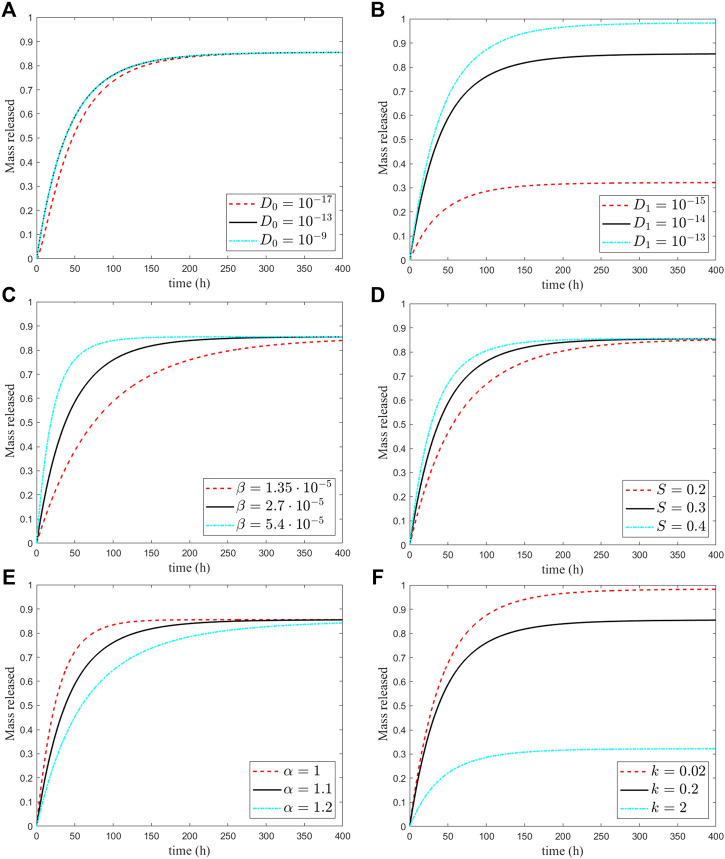
Sensitivity of the release curves to the six model parameters varied with respect to their reference values in Table 1. The black curve refers to the reference values, the red curve to a smaller value, and the blue to a greater value (cfr. tables 1.2).

Despite the high variation of $D_{0}$ , the sensitivity of the drug release is quite low. This is expected because the shell acts as a barrier that hinders mass diffusion, irrespective of the value of the diffusivity in the core (Figure 7A).

In the second set of simulations, we consider the variation of the diffusivity of the shell over $D_{1}^{*}$ , being in the range:

$$\frac{D_{1}}{D_{1}^{*}}=\;0.1-10.$$ (4.5)

The sensitivity to the outer layer diffusivity is more significant and suggests that most of the release characteristics are controlled by shell material properties. In addition, a reduced mobility translates into a longer permanence of drug in the shell, which passes on a stronger binding reaction (Figure 7B). In summary, the release from the MP is diffusion-limited from the lower value of the coating shell that plays a major role and guarantees sustained release. The corresponding changes in release times are reported in Table 2.

**TABLE 2:** Parameter variations over the reference case and their influence on the release time *t*_*r*_.

Parameter ratio	Value	Release time *t*_*r*_ (h)
Reference case (*)	Table 1	233
$D_{0}/D_{0}^{*}$	$10^{-4}-10^{4}$	245–233
$D_{1}/D_{1}^{*}$	0.1−10	233–232
$S/S^{*}$	0.66−1.33	312–178
$\beta /\beta^{*}$	0.5−2	466–119
$k/k^{*}$	0.1−10	233–234
$\alpha /\alpha^{*}$	0.9−1.09	124–334

#### 4.1.2 Influence of the Dissolution

The dissolution process in the core is controlled by the parameters *S* and *β* (Table 2). It turns out that reducing these parameters, and more markedly *β*, results in an impedance of drug dissolution and increasing of releasing time. On the other hand, increasing the values of S and β accounts for an initial burst release of the drug, which may be beneficial when a quick delivery, instead of a sustained and delayed release, is required (Figures 7C,D). An opposite trend has the exponent *α* that through the shape of the dissolved particles and the exposed surface, it controls the amount of the undissolved available drug (Figure 7E). The sensitivity of the releasing time turns out to be very high (Table 2).

#### 4.1.3 Influence of the Binding Rate in the Shell

We first consider one order of magnitude variation oscillating of the parameter k:

$$\frac{k}{k^{*}}=\;0.1-10$$ (4.6)

that corresponds to a reduced/augmented binding rate, respectively, with a different percentage of mass retained in the shell and never released (Figure 7F). The correspondent drug released percentages are 98 and 32% and with a release time unchanged (Table 2).

#### 4.1.4 Influence of the Shape/Curvature

**FIGURE 8:**
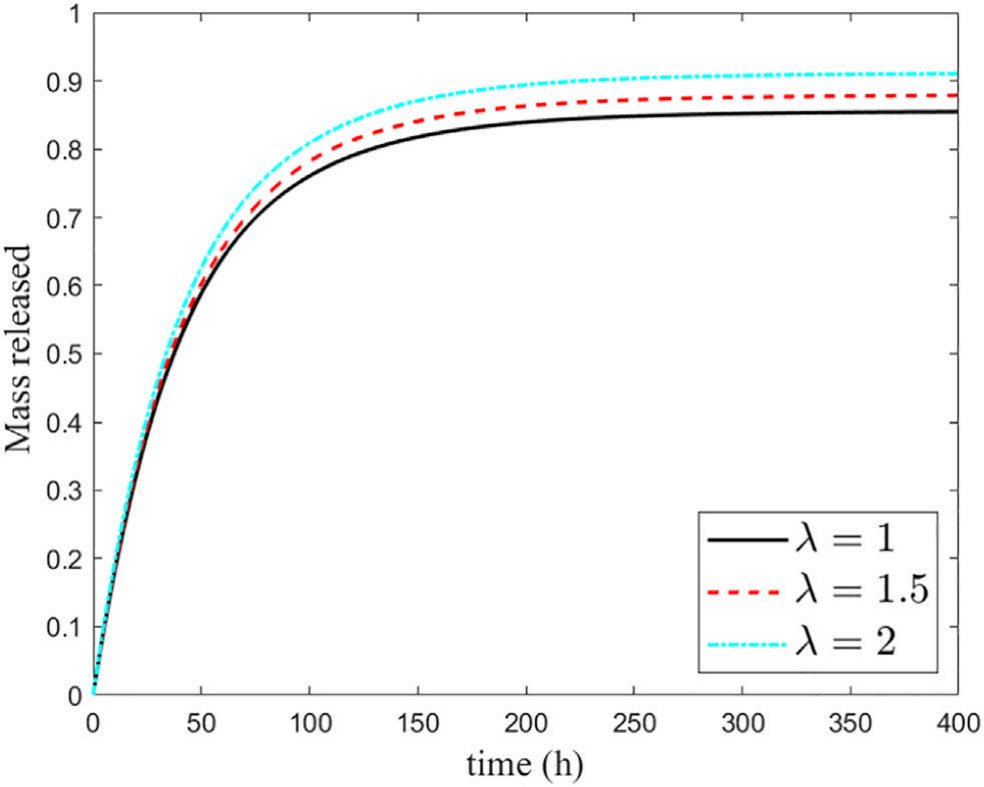
Sensitivity of the release curves to ellipse eccentricity *λ*.

Although the effect of particle surface chemistry and of the size on the delivery of the drug has been widely investigated, a lack of knowledge exists on the influence of the shape of the MP on drug release. Among a variety of irregular configurations, we have analyzed the sensitivity of the release with respect to the varying curvature, in contrast with its constant value of the sphere. For this purpose, a 2D ellipse with different eccentricities has been considered to be a perturbation of the original cross-section circular geometry (Pontrelli et al., 2020). To keep the same volume of the core and the shell, the shell thickness should be reduced to an amount that scales with eccentricity, resulting in a minor effect of the drug retained in it. As a result, an increased percentage of drugs released is reported, with the release time unchanged (Figure 8).

## 5 Discussion

LbL MPs can be easily fabricated by the alternate deposition of charged polymers adsorbed in the multilayer coating onto a spherical core that can be removed to obtain a final bare polymeric particle (Scheffler et al., 2020). Into the solid or hollow core and within the nanolayers, the payload can be entrapped acting as a bio-compound, reporter, or sensor (Tong et al., 2012). Works in literature report that with this LbL-based procedure, it is possible to obtain MPs with dimensions ranging from few to 10–15 μm for releasing, in a controlled and localized manner, a payload for applications in cancer therapy (e.g., chemotherapeutics, photosensitizers, and siRNA), treatment of infectious diseases (e.g., antibiotics and Ag nanoparticles), and cellular engineering (e.g., antigens, peptides, and growth factors) (Tong et al., 2012). In order to create a better design of MPs that can efficiently release the drug payload in the required time for a specific application, the setup of a computational *in silico* model can help.

In our study, we develop a mathematical model consisting of a few assumptions and validated by the experimental data from *in vitro* dissolution tests. Significantly, we have proven that a mechanism of dissolution–diffusion-reaction fitted well the experimental drug release data, and the different recognized parameters of the model point to a complex relationship of drug kinetics with various parameters. The strategy proposed in this work to computationally identify these parameters from experimental *in vitro* data is very beneficial because once calibrated, this model can be utilized in a predictive manner to decrease the number of experiments, and with additional adjustments, can also be associated with *in vivo* experimental results. It is shown how the model parameters influence the entire process of drug release. Therefore, according to the specific application, it is important to identify the set of parameters to be able to ensure a better uniform and prolonged release and the other values that cause a localized peaked distribution followed by a quicker decay. Among these, the coating shell diffusivity provides substantial resistance to the mass flux. This resistance needs to be appropriately tuned to selectively allow the release of the drug molecules, keeping them in the effective therapeutic range without dropping below an insufficient amount or exceeding the toxic dose. The outcomes can be used to evaluate if the drug payload targets the desired tissues at a suitable rate and to tune the required dose load influenced by the multiple shells for a prolonged time. Compared with other existing single-layer models, our proposed work represents an easy tool to predict accurately the release of a drug from an LbL MP that can be beneficial to the design and development of new drug delivery platforms. Finally, the versatility of the proposed model can be also applied to particles functionalized with LbL assembly for different biomedical applications including, for example, tissue engineering and theranostics (Wang et al., 2018). Particularly, this model can help investigate the release of a combination of chemotherapeutic drugs from the core and/or the shell to understand the optimal release kinetics required for fighting cancer cells.

## 6 Conclusion

Although interesting progress in the design of innovative and smart drug release carriers has been made, the specific properties and the manufacturing of functionalized delivery systems remains a great challenge. Mathematical modeling has gained recently extreme importance as an alternative powerful tool to describe drug delivery, and researchers are making a great effort to understand deeper the elution mechanism. This is not fully understood and can be affected by various combined chemical and physical features. In our study, a mechanistic model to investigate the drug release from an LbL functionalized MP is proposed by considering a minimum number of physical assumptions. Numerical results show the role and the importance of the parameters on drug kinetics and demonstrate how the material parameters correlate with the release rate. The experimentally supported outcomes offer a new understanding of the drug mass transfer and of the influence of different parameters, such as the particle shape and the multilayer resistance, on the mechanism of drug release in any release medium including cell culture medium (i.e., DMEM and F12). Thus, by presenting the correlation between properties of the material transport and numerous variables, our model can be utilized to find and optimize the processing parameters that ensure a controlled drug delivery within a certain time. The extension to the surface erodible MPs due to polymer degradation will be considered in a future work. Thus, the proposed model will offer important support in the design of proper particles for achieving a therapeutic release rate by using a systematic strategy with a limited number of experiments.

## Data Availability

The raw data supporting the conclusions of this article will be made available by the authors, without undue reservation.
