# Supplementary material for: Characterization of the Shells in Layer-By-Layer Nanofunctionalized Particles: A Computational Study
Source: Front Bioeng Biotechnol. 2022 Jun 30;10:888944. doi: 10.3389/fbioe.2022.888944 (PMC9280187; doi:10.3389/fbioe.2022.888944)
Supplement: Supplementary file 1 [file DataSheet1.pdf]

Supplementary Material

1. Supplementary Methods

1.1 Scanning Electron Microscopy (SEM)

SEM analysis were performed with a JEOL JSM-5600LV Scanning Electron Microscope model. The Calcium Carbonate sample in powder was deposited on the metallic support, sputtered with gold to make it conductive and subsequently analysed at a working distance of 6mm, an operation voltage of 10 kV and at different magnifications. The pictures were analysed through the software ImageJ.

1.2 Zeta Potential measurement

To measure the surface charge of the bare and functionalised MPs, a Zetasizer Nano ZS Instrument (Malvern Panalytical Ltd) was used. The samples were diluted in distilled H_2O (1:10) and the zeta-potential value was calculated as average of three measurements, each obtained after a maximum of 100 runs.

1.3 Fourier Transformed Infrared spectroscopy (FTIR-ATR)

To assess the surface functional groups of the GQDs FTIR-ATR analysis was conducted. Measurements were obtained with a Spectrum Two PE instrument equipped with a horizontal attenuated total reflectance (ATR) crystal (ZnSe) (PerkinElmer Inc., USA). Data are collected in Absorbance mode, with wavenumber values ranging from 4000 cm^{-1} to 550 cm^{-1}. Each spectrum was the result of the average of 16 scans with 4 cm^{-1} resolution.

1.4 *In vitro* drug release tests

Functionalised drug-loaded MPs were weighted priorly and then immersed in glass vials containing 5 mL of Phosphate Buffered Solution (PBS; pH 7.4, Sigma–Aldrich). The vials were sealed to minimise the changes in the initial pH and incubated at 37 °C without stirring. The blank control ($CaCO_3$ without MT incorporation) was analysed under the same experimental conditions. The medium was withdrawn at different time points for the measurement and replaced with fresh buffer. UV–Vis spectrophotometer (Lambda 2S Perkin Elmer) was used to determine the drug content using maximal absorption peaks for MT at 320 nm. Six replicates were measured, and the results were averaged with standard deviation. The initial content of the MT drug was measured by UV–Vis after dissolution in Ethylenediaminetetraacetic Acid (EDTA, Sigma–Aldrich).

2. Supplementary Figures

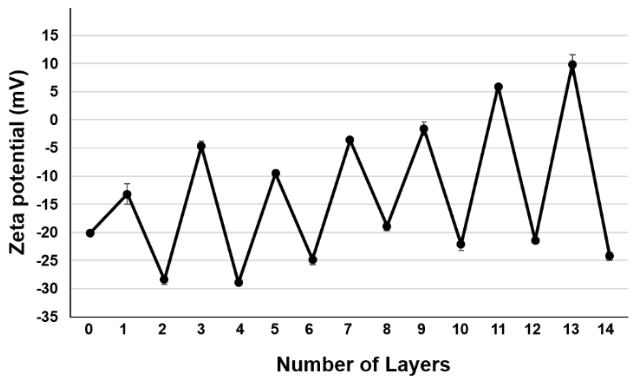

Supplementary Figure S1. ζ-potential measurements as a function of the layer number: layers of odd numbers have PAH as the outermost layer; layers of even number have PSS as the outermost layer (layer zero is the $CaCO_3$ core). Each data point represents mean ± standard deviation of 5 tested samples.

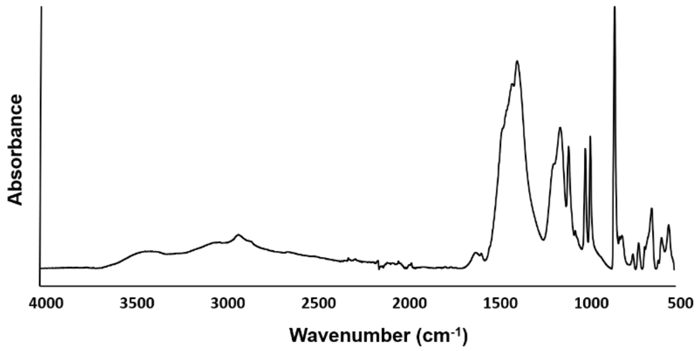

Supplementary Figure S2. ATR infrared spectra of the CaCO3 microparticles coated with 14 nanolayers by LbL assembly.
